# Study on Pore Water Pressure Model of EICP-Solidified Sand under Cyclic Loading

**DOI:** 10.3390/ma17194800

**Published:** 2024-09-29

**Authors:** Gang Li, Yu Li, Xueqing Hua, Jia Liu, Shasha Yang, Yao Zhang

**Affiliations:** 1 Shaanxi Key Laboratory of Safety and Durability of Concrete Structures, Xijing University, Xi’an 710123, China; 2 Guangyuan Natural Gas Co., Ltd., Guangyuan 628000, China; 3 School of Petroleum Engineering and Environmental Engineering, Yan’an University, Yan’an 716000, China

**Keywords:** EICP, sand soil, liquefaction, cyclic stress ratio, pore pressure model

## Abstract

Under traffic load, earthquake load, and wave load, saturated sand foundation is prone to liquefaction, and foundation reinforcement is the key measure to improve its stability and liquefaction resistance. Traditional foundation treatment methods have many problems, such as high cost, long construction period, and environmental pollution. As a new solidification method, enzyme-induced calcium carbonate precipitation (EICP) technology has the advantages of economy, environmental protection, and durability. Through a triaxial consolidated undrained shear test under cyclic loading, the impacts of confining pressure (*σ*_3_), cementation number (*P*_c_), cyclic stress ratio (*CSR*), initial dry density (*ρ*_d_), and vibration frequency (*f*) on the development law of pore water pressure of EICP-solidified sand are analyzed and then a pore water pressure model suitable for EICP-solidified sand is established. The result shows that as *σ*_3_ and *CSR* increase, the rise rate of pore water pressure of solidified sand gradually accelerates, and with a lower vibration number required for liquefaction, the anti-liquefaction ability of solidified sand gradually weakens. However, as *P*_c_, *ρ*_d_, and *f* rise, the increase rate of pore water pressure of solidified sand gradually lowers, the vibration number required for liquefaction increases correspondingly, and its liquefaction resistance gradually increases. The test results are highly consistent with the predictive results, which show that the three-parameter unified pore water pressure model is suitable for describing the development law of A-type and B-type pore water pressure of EICP-solidified sand at the same time. The study results provide essential reference value and scientific significance in guidance for preventing sand foundations from liquefying.

## 1. Introduction

Affected by traffic loads, earthquake loads, and wave loads, the pore water pressure in saturated sand rises sharply, while the effective stress decreases rapidly to zero, causing the shear strength of the sand to lower [1], resulting in liquefaction phenomena such as water spraying [2], sand emitting [3], and subsidence [4], which lead to serious engineering accidents. Foundation treatment is the key measure to improve the stability and anti-liquefaction ability of foundation. At present, the commonly used liquefaction treatment methods include the soil replacement cushion method [5], reinforcement method [6], and chemical reinforcement method [7], etc., which usually have problems such as high cost, long construction period, and environment pollution. Thus, the most important thing is to find an efficient [8], environmentally friendly [9], cost-effective [10], and durable [11] solidification method. Microbially induced carbonate precipitation (MICP) and enzyme-induced carbonate precipitation (EICP) are new curing methods developed in recent years that have the characteristics of being highly efficient, environmentally friendly, and durable when compared with traditional methods of solidified sandy soil [12,13,14]. It mainly catalyzes urea hydrolysis via enzymes to generate ammonium ions and carbonate ions. Ammonium ions can provide an alkaline environment for the solution, while carbonate ions combine with calcium ions to generate calcium carbonate, which can improve strength and reduce permeability by rapidly cementing soil particles [15,16,17,18,19,20]. Compared with MICP technology, EICP technology does not require a lengthy bacterial culture process and has no biosafety issues [21]. Moreover, enzymes are much smaller in size than bacteria [22].

Sun et al. [21] discovered that the optimal urease (1.0 mol/L) and complex strength (4.9 MPa) of specimens in the EICP study were 1.56 times that of constant humidity specimens. Thomas et al. [23] discovered that the soft oil was treated with nano silica, bioenzyme, and cement under dynamic load, and through a cyclic triaxial test, the nano dioxide effect was very high for improving the stability of sandy oil under the conditions of biologic enzyme and cement. Saif et al. [24] discovered that the urease activity and calculation carbonate formation rate were increased with increasing temperature when urease and curing time were fixed. When the concentration of the reactant contacts a certain concentration, the reaction rate and prevention amount will be reduced. If the curing time is prolonged, the precession ratio of calcium carbonate can reach 100%. Shen et al. [25] discovered that EICP, EICP-BF (Basalt fiber), and EICP-PVAC (Polyvinyl acetate emulsion) can promote the surface strength (SS) and capacity of sandy soil. The sand treated with EICP-0.5% BF, EICP-30 g/L PVAC, and EICP-50 g/L PVAC has higher erosion resistance. Zhu et al. [26] discovered that the liquefaction persistence of sand is directly proportional to the content of slag power. Under the cyclic triaxial test, with a cyclic stress ratio of 0.2, the number of liquefaction cycles of the solidified soil has risen by 30 instead of the unsolidified soil, which greatly increments the stability of sand. Li et al. [27] discovered that after 30 days of seawater erosion, the strength of the specimens retained 35.19% of the starting strength. The weight was reduced by 6.69%. The test results show that the specimen retains its integrity despite being eroded by sea water. Wang et al. [28] discovered that combining polyacrylic acid (PAA) and EICP can promote sandy soil strength. When the slope of the surface strength lines of EICP-PAA-treated specimens is *k* = 13.434, 14.002 and 14.186, the surface strength of EICP-PAA-treated specimens is higher than that of EICP (*k* = 14.271) solidified specimens. Wu et al. [29] discovered that the urease activity incremented from 2.95 U/mL to 5.39 U/mL and the cementing solution incremented from 0.25 M to 0.75 M, which could improve the unconfined comprehensive strength of the specimen sand. Cui et al. [30] investigated the curing action of EICP combined with xanthan gum on sand. It was found that the permeability coefficient of 2% xanthan gum was only 65.4% of that of the pure EICP treatment, and the addition of xanthan gum would accelerate the formation of CaCO_3_ and increase the vision between sand parties. Alotaibi et al. [31] conclude that, in terms of soil stabilization, the EICP soil treatment has nearly a 90% lower abiotic depletion potential and a 3% reduction in global warming potential compared to Portland cement.

EICP technology can greatly enhance the strength of the soil and decrease its permeability, but its effectiveness in improving the soil’s liquefaction resistance still needs to be validated. So, it is particularly important to clarify the development law of the pore water pressure of solidified soil under cyclic loading. Based on the triaxial consolidated undrained shear test of EICP-solidified sand under cyclic loading, the effects of confining pressure, cementation number, cyclic stress ratio, dry density, and vibration frequency on the development of pore water pressure of EICP-solidified sand were analyzed, and the pore water pressure model suitable for EICP-solidified sand was established and verified. The study findings hold significant theoretical value and scientific importance for promoting EICP technology in the prevention and control of sand liquefaction.

## 2. Materials and Methods

### 2.1. Test Materials

**Figure 1:**
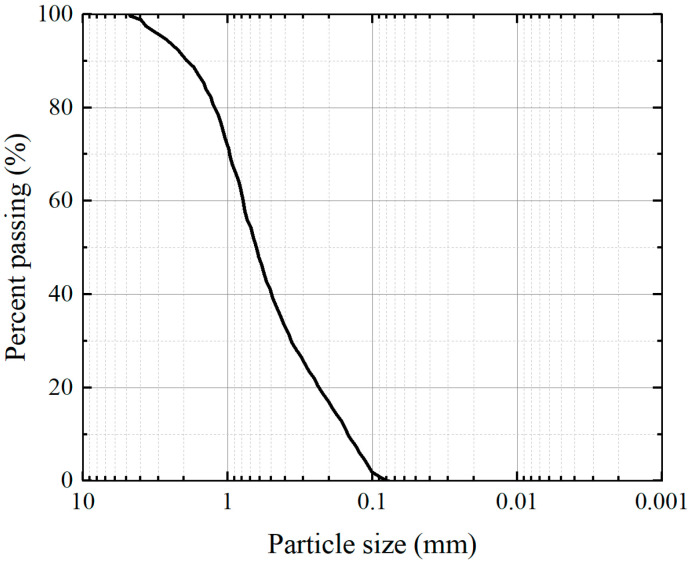
Particle grade curve.

The test enzyme is soybean enzyme extract, and the soybean is produced in Yilan County Sanxing Ecological Agriculture Co., Harbin, China, which is a non-transgenic product with round and full particles. During the experiment, the soybean was dried and ground into powder, mixed with deionized water after passing through a 0.150 mm sieve, stirred in a magnetic stirrer for 30 min, centrifuged in a centrifuge at a speed of 3000 r/min for 30 min, and then the supernatant was extracted, which was enzyme solution. The enzyme solution prepared by mixing 100 g soybean powder with 1000 mL water was used for the follow-up test. The urea and anhydrous calcium chloride are purchased from China National Pharmaceutical Group Chemical Reagent Company, Shanghai, China. The cementing solution is the mixture of urea and anhydrous calcium chloride, which is prepared 1:1 (equal concentration and equal volume). The standard sand used in the test is China ISO standard sand (GB/T 50123-2019) [32], which is purchased from Xiamen Aisiou Standard Sand Co., Ltd., Xiamen, China. The standard sand grading curve is displayed in Figure 1. The physical property index of standard sand is shown in Table 1.

### 2.2. Sample Preparation

**Figure 2:**
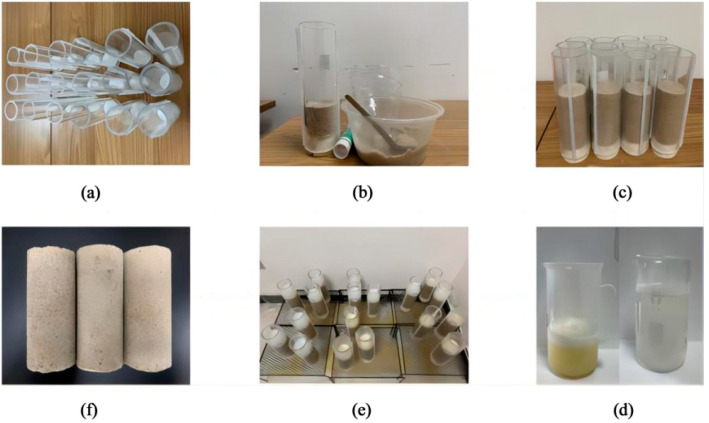
Specimen preparation flow chart: (**a**) specimen mold; (**b**) inject the sand; (**c**) sand specimen; (**d**) enzyme extraction; (**e**) standing specimen a certain time; (**f**) test specimens.

The Plexiglas tube with an internal diameter of 50 mm and a height of 200 mm is chosen for the sample mold. The mold is half-open for taking out the solidified specimen and is fixed by hot melt adhesive when used. The triaxial specimen is 50 mm in diameter and 100 mm in height. To avoid leakage during pouring, the bottom of the mold is sealed with a rubber plug. Based on the set dry density, the specimen is loaded in 4 layers. The standard sand is evenly and slowly sent into the mold by funnel and drug spoon, and the sand column is compacted by compaction hammer. After the specimen is placed, a layer of filter paper is laid on top to reduce the impact disturbance of the pouring liquid. In the process of pouring, 40 of mL enzyme was first poured into the mold, and then stood for 30 min after enzyme grouting until enzyme fully penetrated into the pores of sand. Then, 40 mL of cementing solution was poured. After pouring, the specimen was left for 24 h and air-dried at normal temperature. The specimen preparation process is shown in Figure 2.

### 2.3. Test Methods

To analyze the cumulative characteristics of pore water pressure of EICP-solidified sand under cyclic loading, a triaxial consolidated undrained shear test was carried out by using DYNTTS dynamic triaxial soil testing machine (manufactured by Earth Products China Limited, Hong Kong, China). The influence of confining pressure (*σ*_3_), cementation number (*P*_c_), cyclic stress ratio (*CSR*), initial dry density (*ρ*_d_), and vibration frequency (*f*) on the pore water pressure ratio (*r*_u_) was considered in the test process. *P*_c_ represents the number of injected cycles with enzyme solution and cementing solution. *CSR* [33,34] is defined as the ratio of the amplitude of the applied cyclic stress to two of the effective confining pressures after consolidation. The *r*_u_ [35] is the ratio between the accumulated excess pore water pressure and the confining pressure. The test scheme is displayed in Table 2. Based on the geotechnical test method standard (GB/T 50123-2019) [32], the specimen is first put into a vacuum saturation cylinder for saturation for more than 10 h and then taken out and loaded into a pressure chamber for step-by-step back pressure saturation. Once the pore water pressure coefficient *B* is greater than 0.99, the specimen is saturated. After the specimen is saturated, the effective confining pressure is applied to consolidate for more than 60 min, and the consolidation is completed after the drainage volume has no change. Stress control is adopted in the test, sinusoidal wave load is applied axially, and the number of cycles (*N*) is 10,000. When the pore pressure reaches the effective confining pressure, the test is stopped. The 108-group tests were carried out, and repeated tests were set for each group of specimens.

Based on relevant research [36,37,38,39,40,41], the existing pore water pressure prediction model was fitted and analyzed with test data, and a new pore water pressure model for EICP-solidified sand was established to verify its feasibility. Table 3 summarizes the existing prediction models of the pore water pressure of sand.

## 3. Results and Discussion

### 3.1. Analysis of the Influence of Confining Pressure on Pore Water Pressure

**Figure 3:**
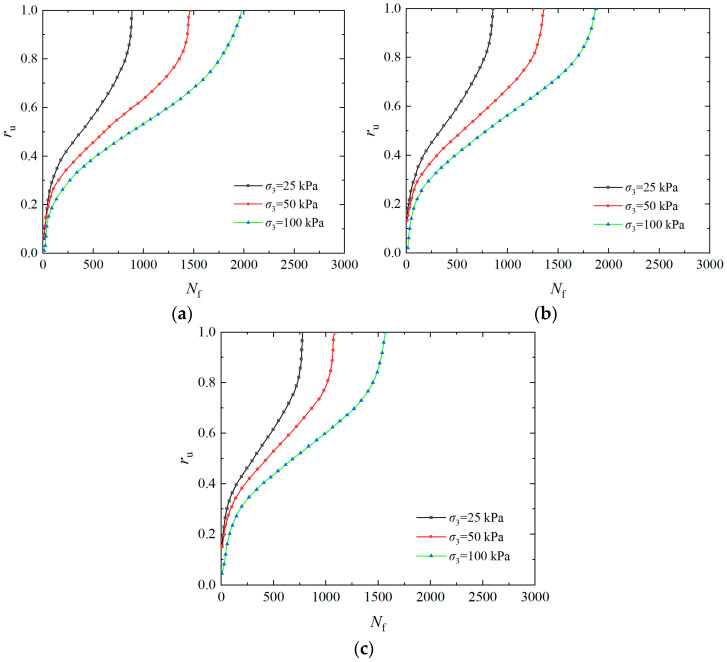
Influence of *σ*_3_ on pore water pressure of EICP-solidified specimen: (**a**) *CSR* = 0.25; (**b**) *CSR* = 0.30; (**c**) *CSR* = 0.35.

The *σ*_3_ generally rises with increasing soil depth, so it has an important influence on the pore water pressure accumulation. Figure 3 shows the curves of the over pore pressure ratio of the solidified specimen with a vibration number under *ρ*_d_ of 1.6 g/cm^3^, *P*_c_ of 2, *f* of 1 Hz, and *CSR* of 0.25, 0.30, and 0.35, respectively. The pore water pressure varies in three stages as the vibration number rises, as seen in Figure 3: in the instantaneous increase section, the curve is convex (pore pressure increases rapidly); in the middle gentle section, the curve is nearly linear (pore water pressure rises slowly); and in the instantaneous liquefaction section, the curve is concave (pore water pressure increases rapidly). When the *CSR* is 0.25, the *r*_u_ is 0.6 under *σ*_3_ of 100 kPa, the cumulative curve of pore water pressure is steep, and the vibration number is less when liquefaction is reached. However, the *r*_u_ of the specimen under *σ*_3_ of 25 kPa is 0.4, the pore pressure curve is slow, and the vibration number is the highest when liquefaction is reached. It can be seen that as the *σ*_3_ increases, the failure vibration number of the specimen decreases, while the growth rate of pore water pressure accelerates, and the pore water pressure curve shows an increasing trend from slow to steep, indicating that the liquefaction resistance of the solidified specimen gradually decreases [42]. The primary reason for this phenomenon is that the *σ*_3_ has a significant effect on dynamic stress. Under significant dynamic loading, the soil structure is largely destroyed. The soil stress is entirely born by the pore water pressure, and effective stress gradually decreases to zero, which makes liquefaction more likely to occur.

### 3.2. Analysis of the Influence of Cementation Number on Pore Water Pressure

**Figure 4:**
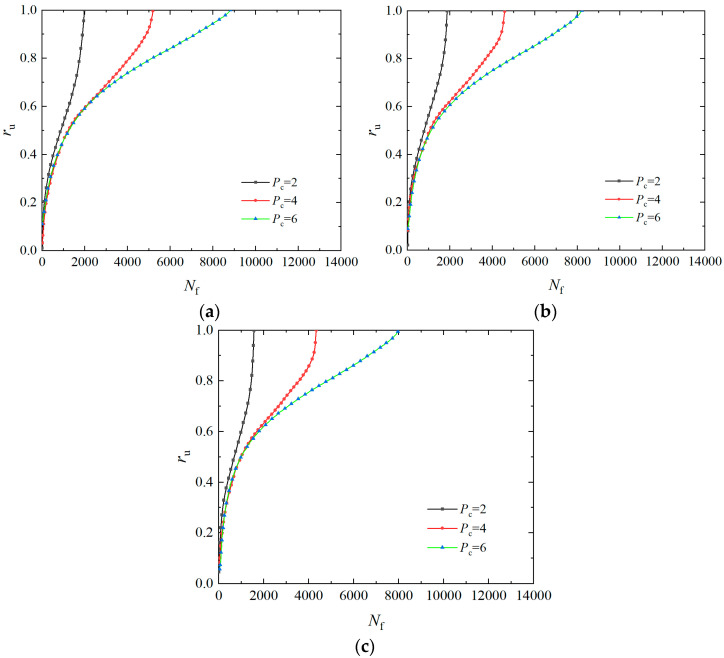
Effect of *P*_c_ on pore water pressure of EICP-solidified specimen: (**a**) *CSR* = 0.25; (**b**) *CSR* = 0.30; (**c**) *CSR* = 0.35.

To study the effect of the *P*_c_ on the accumulation law of pore water pressure of EICP-solidified sand, Figure 4 summarizes the curves of the over pore water pressure ratio with the vibration number at *ρ*_d_ of 1.6 g/cm^3^, *σ*_3_ of 25 kPa, and *f* of 1 Hz, and *CSR* of 0.25, 0.30, and 0.35, respectively. Seeing Figure 4, the pore water pressure curve of solidified specimens changes from steep to slow as the *P*_c_ rises. The velocity of the increase in pore water pressure decreases gradually, and the vibration number required for failure increases gradually. This indicates that the rise in *P*_c_ contributes to enhancing the liquefaction resistance of EICP-solidified sand. The primary factor contributing to this phenomenon is that, as the *P*_c_ rises, CaCO_3_ crystals produced by EICP mineralization gradually increase. The CaCO_3_ crystals can efficiently occupy the pores between sand particles, cementing sand particles together, thus improving the overall mechanical properties of the treatment sands. Under vibration loading, the solidified soil skeleton and pore water pressure bear the dynamic stress together, which leads to the liquefaction of the specimen.

### 3.3. Analysis of the Influence of Cyclic Stress Ratio on Pore Water Pressure

**Figure 5:**
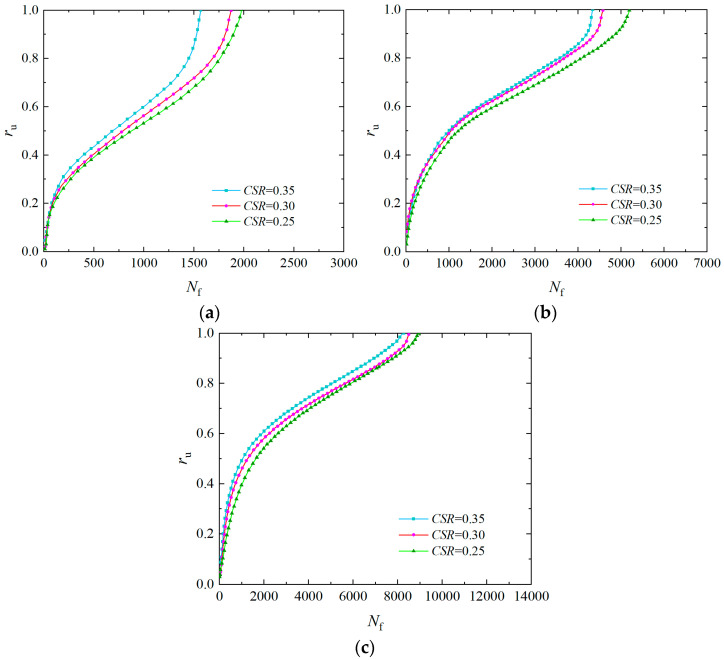
Effect of *CSR* on pore water pressure of EICP-solidified specimen: (**a**) *P*_c_ = 2; (**b**) *P*_c_ = 4; (**c**) *P*_c_ = 6.

To study the effect of the *CSR* on pore water pressure accumulation characteristics, Figure 5 shows the curve between the excess pore water pressure ratio and vibration number when the *ρ*_d_ is 1.6 g/cm^3^, the *σ*_3_ is 25 kPa, the *f* is 1 Hz, and the *P*_c_ are 2, 4, and 6. For the number of the same vibrations, the higher the *CSR*, the higher the corresponding the *r*_u_. The greater the *CSR*, the fewer vibrations number are required for the liquefaction of the specimen, which indicates that the rise in the *CSR* accelerates the liquefaction trend in the specimen. The reason is that the rise in the *CSR* will exert stronger shear stress in a short time, which will lead to sand particles being rearranged, enhance the connectivity of pores in the sand, cause the rapid rise in the pore water pressure, and make the liquefaction trend in specimens more obvious [43]. When *P*_c_ is 6, the vibration number required for specimen liquefaction does not change significantly with the increase in the *CSR*. The main reason is that when *P*_c_ is 6, the EICP mineralization reaction generates a large amount of CaCO_3_ to fill the pores, cementing sand particles, increasing the cohesion and the shear strength of sand, and improving the liquefaction resistance of the specimen [44].

### 3.4. Analysis of the Influence of Dry Density on Pore Water Pressure

**Figure 6:**
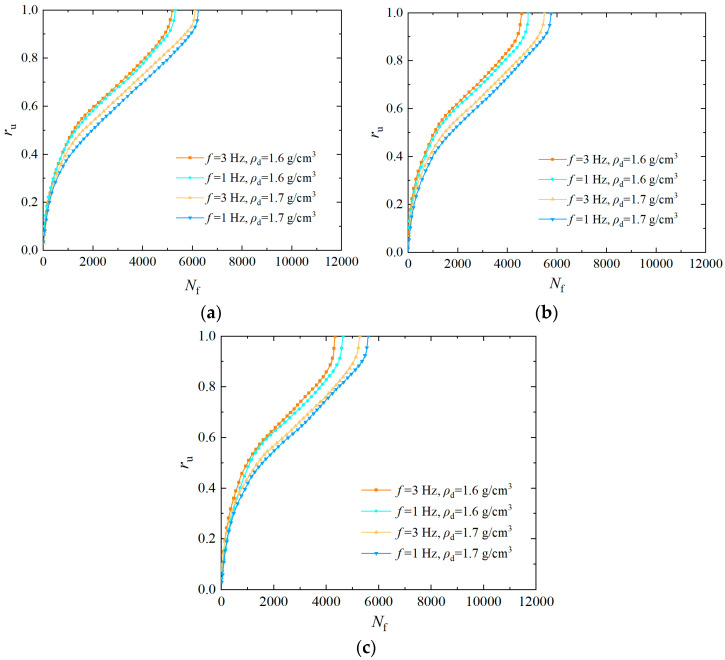
Effect of *ρ*_d_ on pore water pressure of EICP-solidified specimen**:** (**a**) *CSR* = 0.25; (**b**) *CSR* = 0.30; (**c**) *CSR* = 0.35.

The *ρ*_d_ affects the liquefaction resistance of EICP-solidified sand. Figure 6 summarizes the curves of the over pore pressure ratio versus the vibration number at *σ*_3_ of 25 kPa, *P*_c_ of 4, and *CSR* of 0.25, 0.30, and 0.35, respectively. In seeing Figure 6, the cumulative curve of the pore water pressure of the specimen is steep at *ρ*_d_ of 1.6 g/cm^3^, and the vibration number required for liquefaction is less. However, the cumulative curve of the pore water pressure of the specimen is smooth, and the vibration number required for liquefaction rises when the *ρ*_d_ is 1.7 g/cm^3^. This indicates that as the *ρ*_d_ of the specimen increases, the rate of the growth of the pore water pressure decreases and the vibration number required for failure increases correspondingly [45]. This phenomenon occurs because the contact points between sand particles increase, and the pores between particles decrease as the *ρ*_d_ of the specimen rises. CaCO_3_ crystals produced by EICP mineralization can better cement sand particles, which leads to the decrease in pore volume and the deterioration of the permeability of the specimen. Under cyclic loading, the pore water pressure rises slowly, which delays the liquefaction process of specimens.

### 3.5. Analysis of the Influence of Frequency on Pore Water Pressure

**Figure 7:**
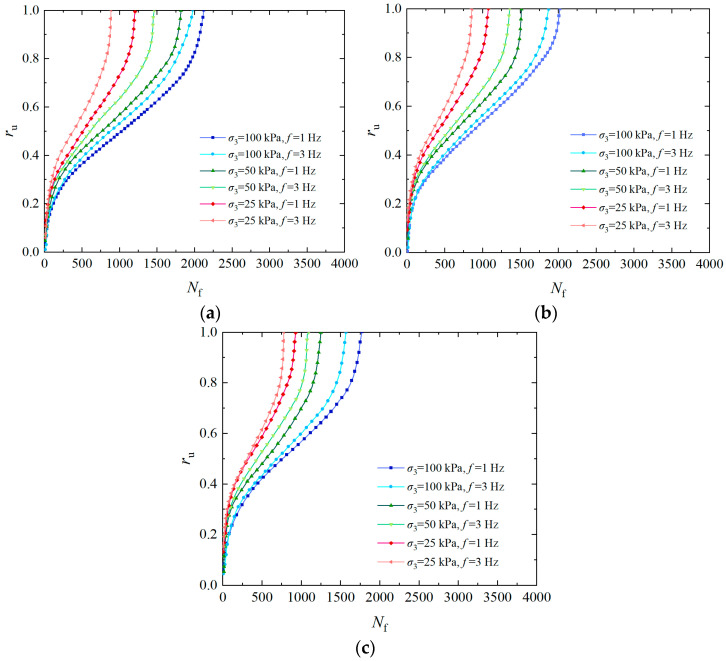
Influence of *f* on pore water pressure of EICP-solidified specimen: (**a**) *CSR* = 0.25; (**b**) *CSR* = 0.30; (**c**) *CSR* = 0.35.

The *f* directly reflects the moving speed of the dynamic load and vibration number, affecting the liquefaction resistance of EICP-solidified sand. Figure 7 summarizes the curves of the over pore pressure ratio versus the vibration number at the *ρ*_d_ is 1.6 g/cm^3^, the *P*_c_ is 2, and the *CSR* are 0.25, 0.30, and 0.35, respectively. Seeing Figure 7, the *r*_u_ of the specimen reduces gradually as the rise in *f* at the vibration number is constant. The vibration number required for liquefaction of specimen gradually rises with the improvement in *f* when the *r*_u_ is constant. That is, as *f* increases, the rise speed of pore water pressure of the specimen gradually reduces and the vibration number required for liquefaction gradually improves. The reason is that the pore pressure transfer is affected by the drainage channel formed by the pore penetration of the soil skeleton. Under high-frequency cyclic loading, the dislocation between sand particles will lead to the reconstruction of the drainage channel, and the reconstructed channel will have a blocking effect due to the action of small particles, thus reducing the rate of pore water pressure transmission and improving the liquefaction resistance of solidified specimen.

## 4. Pore Pressure Model of EICP-Solidified Standard Sand

### 4.1. Analysis of Pore Pressure Development Model

**Figure 8:**
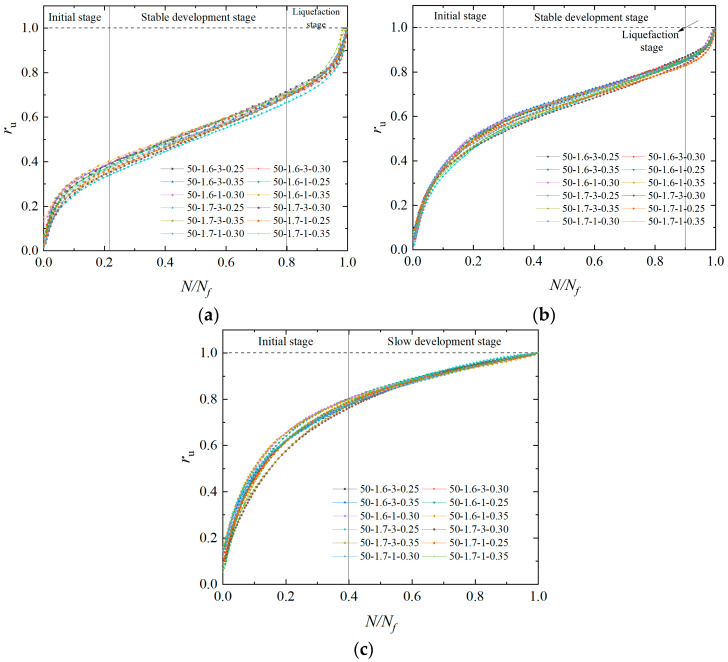
Development pattern of pore water pressure of EICP-solidified specimen: (**a**) *P*_c_ = 2; (**b**) *P*_c_ = 4; (**c**) *P*_c_ = 6.

Figure 8 summarizes the evolution pattern of the pore water pressure of the EICP-solidified sand at a *P*_c_ of 2, 4, and 6. Based on the development pattern of the pore water pressure in EICP-solidified standard sand, the *P*_c_ has a significant impact on the pore water pressure, while *ρ*_d_ has a lesser effect. Generally, the modes of developing pore water pressure can be classified into two types: Type A and Type B. The A-type pore water pressure development model can be divided into three stages: in the instantaneous rise stage, the curve is convex (pore water pressure increases rapidly); in the middle gentle section, the curve is nearly linear (pore water pressure increases slowly); and in instantaneous liquefaction section, the curve is concave (pore water pressure increases rapidly). The B-type pore water pressure development model can be divided into two stages: the instantaneous increasing stage and the intermediate gentle stage. When the *P*_c_ are 2 and 4, the development pattern of pore water pressure of EICP-solidified sand is mainly type A, while, when the *P*_c_ is 6, the development pattern is mainly type B. The reason is that fewer CaCO_3_ crystals are generated by EICP mineralization when the *P*_c_ is lower (2 and 4), which mainly act to cover and fill pores, but do not play an effective role in cementing sand particles. When the vibration number reaches a certain value, the skeleton between sand particles is destroyed, and the dynamic stress is transferred to pore water pressure, leading to the sudden rise in the pore water pressure and liquefaction. When the *P*_C_ is greater, CaCO_3_ crystals can cement sand particles together and enhance the skeleton action of sand particles. With the increase in the vibration number, the connection between sand particles has not been broken, but some sand particles exhibit dislocation displacement, which leads to pore communication, and pore water pressure reaches the liquefaction state in the steady growth stage [46].

### 4.2. Establishment and Verification of Pore Water Pressure Model of Solidified Sand by EICP

**Figure 9:**
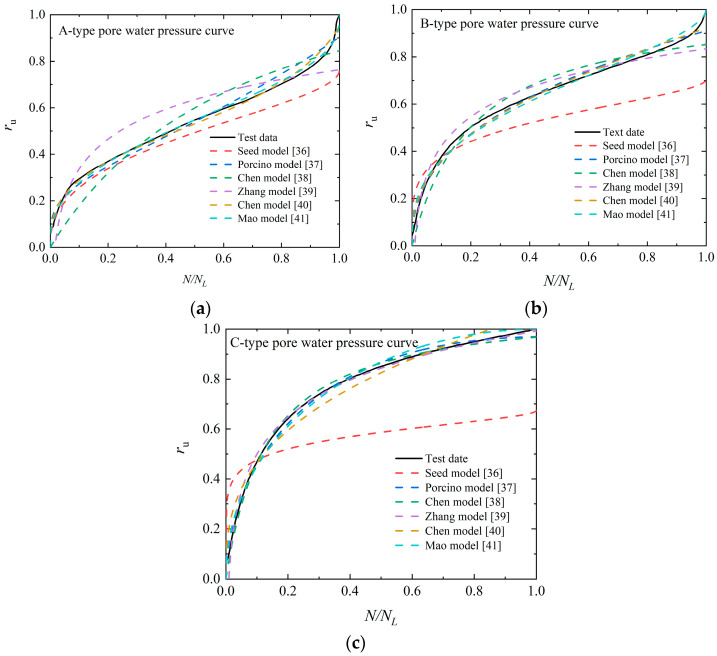
Comparison between pore pressure test and model prediction results: (**a**) *P*_c_ = 2; (**b**) *P*_c_ = 4; (**c**) *P*_c_ = 6.

To further analyze the evolution law of pore water pressure of EICP-solidified sand, Table 3 summarizes the existing prediction models of pore water pressure of sand. The model presented in Table 3 is utilized for the fitting and analysis of the test data, with the corresponding parameter values provided in Table 4. The comparison between the pore water pressure test and prediction outcomes is summarized in Figure 9. Seeing Figure 9, different pore water pressure prediction models have different prediction results for A-type and B-type pore water pressure development models. For the A-type pore water pressure development model, the predictions from the Porcino model, Chen model, and Mao model align well with the experimental results, and the determinability coefficient (*R*^2^) is over 0.980, while the prediction results of the Seed model are poor. For the B-type pore water pressure development model, the prediction results of the Porcino model, Chen model, Zhang model, Chen model, and Mao model align well with the experimental results, and the *R*^2^ is over 0.971.

Aiming at EICP-solidified sand, to establish a pore water pressure prediction model that can describe the development mode of A-type and B-type pore water pressure simultaneously, a new unified pore water pressure prediction model is established by modifying parameters based on the present study outcomes. Equation (1) is as follows:

(1) $$r_{u}=\frac{u}{u_{f}}=a{\left[1-{\left(1-\frac{N}{N_{f}}\right)}^{b}\right]}^{\frac{1}{c}}$$

where *r*_u_ is pore pressure ratio (dimensionless); *N*/*N*_f_ is the ratio of vibration number (dimensionless); and *a*, *b* and *c* are model parameters (dimensionless).

**Figure 10:**
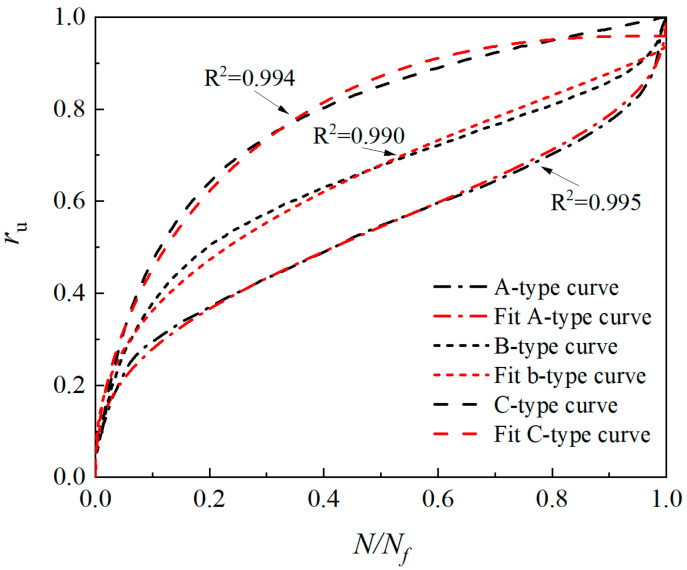
Comparison between prediction and test results of newly built pore water pressure model.

Table 5 lists the pore water pressure model parameters of EICP-solidified sand. The analysis reveals that the power value 1/c has a critical effect on shaping the curve. When the 1/c is small, the power value of the function is big, and the curve presents an S-shape; however, when the 1/c is larger, the power value of the function is smaller, and the curve tends to be hyperbolic. Parameters a and b are associated with the slope and intercept of the curve, respectively. Higher values of a will increase the slope of the curve, whereas higher values of b will increase the intercept of the curve. When the A-type curve transitions to the B-type curve, *b* and *c* parameters increase, while *a* parameters decrease. When transitioning from a B-type curve to a C-type curve, *a* and *b* parameters rise, while *c* parameters decrease. Figure 10 illustrates the predicted outcomes of the newly built pore water pressure model and the experimental results. The test results align well with the predicted results, and the *R*^2^ is over 0.990. This shows that the model can greatly describe the development law of the A-type and B-type pore water pressure of EICP-solidified sand simultaneously. It should be pointed out that the applicable conditions of this model are as follows: for the consolidated undrained shear test under cyclic load and loading sine wave, the *σ*_3_ is 25~100 kPa, the *f* is 1~3 Hz, and the *ρ*_d_ of sand is 1.6~1.7 g/cm^3^; *P*_C_ is 2~6. The failure criterion for the specimen is defined as the *r*_u_ reaches 1.

## 5. Conclusions

The effects of confining pressure (*σ*_3_), cementation number (*P*_c_), cyclic stress ratio (*CSR*), initial dry density (*ρ*_d_), and vibration frequency (*f*) on the development of the pore water pressure of EICP-solidified sand were analyzed through a triaxial consolidated undrained shear test under cyclic loading. Based on the dynamic triaxial experiment outcomes, a pore water pressure model was established to describe the development of A-type and B-type pore water pressure in EICP-solidified sand. The conclusions are as follows:

1. The pore pressure changes in three stages with the increase in the vibration number: in the instantaneous increase section, the curve is convex; in the middle gentle section, the curve is nearly linear; and in instantaneous liquefaction section, the curve is concave. As the *σ*_3_ rises, the failure vibration number of the specimen decreases. The increase in the pore water pressure accelerates, and the pore water pressure curve shows an increasing trend from slow to steep, indicating that the liquefaction resistance of the solidified specimen is gradually weakened.
2. As the increase in *P*_c_, the pore water pressure curve of EICP-solidified specimens, changes from steep to slow, the increasing rate of pore water pressure decreases gradually, and the vibration number required for failure increases gradually, which indicates that the increase in *P*_c_ is helpful to improve the liquefaction resistance of EICP-solidified sand.
3. When the vibration number is the same, the greater the *CSR*, the higher the *r*_u_, and the lower vibration number required for the liquefaction of the specimen, which indicates that the increase in *CSR* accelerates the liquefaction trend in the specimen.
4. With the increase in the *ρ*_d_ of the specimen, the growth rate of the pore water pressure decreases, and the vibration number required when liquefaction failure is achieved increases accordingly.
5. When the vibration number is constant, the *r*_u_ decreases with the increase in vibration frequency. When the *r*_u_ is constant, with the rise in *f*, the increased rate of pore pressure of the specimen gradually decreases, and the required vibration number gradually increases when liquefaction is achieved.
6. The three-parameter unified pore water pressure prediction model is applied to calculate the test results, and the test results align well with the predicted results, which demonstrates that the model is suited to describe the development law of the A-type and B-type pore water pressure of EICP-solidified sand simultaneously.
7. In the future, the struvite precipitation can be considered to recover the ammonia generated during the EICP mineralization reaction process, which can reduce environmental pollution [47]. The enzyme solution and cementing solution can be sprayed on the foundation of saturated loose sand to solidify the sand and improve foundation resistance to liquefaction.

## Figures and Tables

**Table 1:** Physical property index of standard sand.

Specific Gravity *G*_s_	Maximum Dry Density *ρ*_d max_ (g/cm^3^)	Minimum Dry Density *ρ*_d min_ (g/cm^3^)	Effective Diameter *d*_10_ (mm)	Median Diameter *d*_30_ (mm)	Constrained Diameter *d*_60_ (mm)	Coefficient Uniformity *C*_u_	Curvature Coefficient *C*_c_
2.65	1.73	1.46	0.14	0.50	0.90	6.43	1.98

**Table 2:** Dynamic triaxial test scheme.

*σ*_3_ (kPa)	*P*_c_	*CSR*	*ρ*_d_ (g/cm^3^)	*f* (Hz)
25	2	0.25, 0.30, 0.35	1.6, 1.7	1, 3
	4	0.25, 0.30, 0.35	1.6, 1.7	1, 3
	6	0.25, 0.30, 0.35	1.6, 1.7	1, 3
50	2	0.25, 0.30, 0.35	1.6, 1.7	1, 3
	4	0.25, 0.30, 0.35	1.6, 1.7	1, 3
	6	0.25, 0.30, 0.35	1.6, 1.7	1, 3
100	2	0.25, 0.30, 0.35	1.6, 1.7	1, 3
	4	0.25, 0.30, 0.35	1.6, 1.7	1, 3
	6	0.25, 0.30, 0.35	1.6, 1.7	1, 3

**Table 3:** Prediction model of pore pressure.

Number	Pore Pressure Model Formula	Parameter	Formula Source
1	$\frac{u}{u_{f}}=\frac{2}{\pi }\arcsin{\left(\frac{N}{N_{f}}\right)}^{\frac{1}{2\theta }}$	θ = *c*_1_·*FC* + *c*_2_·*D*_r_ + *c*_3_·*CSR* + *c*_4_ *c*_1_, *c*_2_, *c*_3_, *c*_4_—Calculated parameters	Seed et al. [36]
2	$\frac{△u}{{\sigma^{\prime }}_{\mathrm{vo}}}=a{\left(\frac{N_{c}}{N_{f}}\right)}^{b}c^{\frac{N_{c}}{N_{f}}}$	*a*, *b*, *c*—Calculated parameters	Porcino et al. [37]
3	$\frac{u}{u_{f}}=\frac{N/N_{f}}{a+b(N/N_{f})}$	*a*, *b*—Calculated parameters	Chen et al. [38]
4	$\frac{u^{*}}{u_{f}}=1-e^{-\beta \frac{t}{t_{f}}}$	*β*—Calculated parameters *u*_f_—Boundary pore pressure t_f_—Loading time	Zhang et al. [39]
5	$\frac{u}{{\sigma^{\prime }}_{0}}=(1-ma_{s})\frac{2}{\pi }\arcsin{\left(\frac{N}{N_{L}}\right)}^{\frac{1}{2\theta }}$	*α_S_*, *m*, *θ*—Calculated parameters	Chen et al. [40]
6	$\frac{△u}{△u_{f}}={\left[1-{\left(1-\frac{N}{N_{f}}\right)}^{m}\right]}^{\frac{1}{\theta }}$	*m*, *θ*—Calculated parameters	Mao et al. [41]

**Table 4:** Fitting parameter values of pore pressure model.

Curve Type	Seed Model			Porcino Model			Chen Model		
	*β*	*R* ^2^	*a*	*b*	*c*	*R* ^2^	*a*	*b*	*R* ^2^
Type A	1.45	0.840	0.37	0.14	2.29	0.98	0.40	0.96	0.900
Type B	1.58	0.650	0.71	0.46	1.45	0.991	0.51	0.52	0.960
Type C	1.36	0.240	1.02	0.22	0.96	0.995	0.059	1.018	0.995
Curve Type	Zhang Model			Chen Model			Mao Model		
	*a*	*R* ^2^	*a*	*b*	*θ*	*R* ^2^	*m*	*θ*	*R* ^2^
Type A	1.61	0.840	1.631	1.488	3.694	0.992	0.24	2.93	0.994
Type B	2.23	0.950	3.049	0.841	1.819	0.990	0.90	0.60	0.988
Type C	6.02	0.991	7.606	0.013	4.820	0.971	0.93	0.21	0.990

**Table 5:** Parameters of pore water pressure model of EICP-solidified sand.

Curve Type	Model Parameters			
	*a*	*b*	*c*	*R* ^2^
Type A	1.047	0.277	2.552	0.994
Type B	0.946	0.790	2.717	0.990
Type C	0.972	2.683	2.203	0.995

## Data Availability

Data are contained within the article.
